# Supplementary material for: Genetically engineered mesenchymal stem cells as a nitric oxide reservoir for acute kidney injury therapy
Source: eLife. 2023 Sep 11;12:e84820. doi: 10.7554/eLife.84820 (PMC10541176; doi:10.7554/eLife.84820)
Supplement: Figure 1—source data 1. [file elife-84820-fig1-data1.docx]

**Source data 1**

**Synthesis route of the NO-prodrug MGP**.


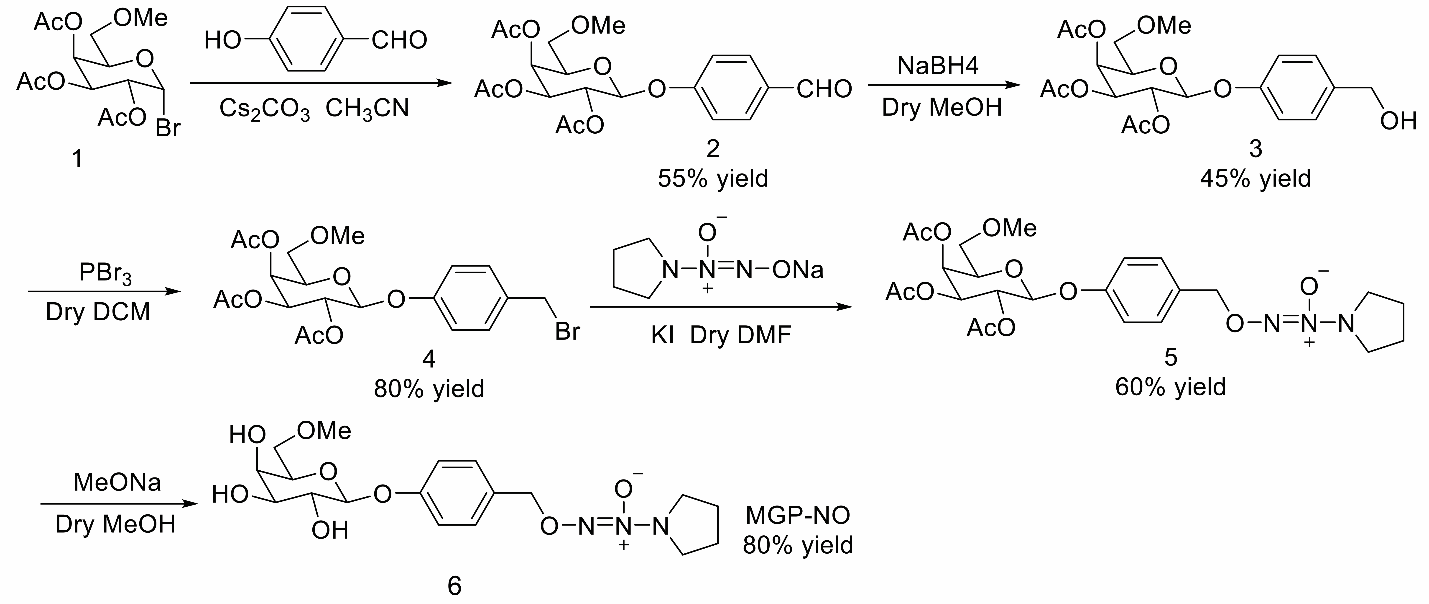


The synthesis route for MGP is performed with five reactions from compound 1 to obtain compound 6, the final product, MGP. The synthesis route for MGP is described below. The following splitting abbreviations were used: s = singlet, d = doublet, dd = doublet doublet, t = triplet, m = multiplet.

*1. Compound 1 ((2R,3R,4S,5S,6R)-2-bromo-6-(methoxymethyl) tetrahydro-2H-pyran-3,4,5-triyl triacetate) was prepared according to our previously reported method* (Nat Chem Biol. 2019;15(2):151-60).

*2. The synthesis of Compound 2*, *(2S,3R,4S,5S,6R)-2-(4-formyl phenoxy)-6-(methoxymethyl) tetrahydro-2H-pyran-3,4,5-triyl triacetate*

To obtain a solution of compound 1 (1 g, 2.2 mmol), 4-hydroxybenzaldehyde (0.42 g, 3.4 mmol) and cesium carbonate (2 g, 6.1 mmol) in dry CH_3_CN (30 mL) were added. The mixture was stirred for 24 h at room temperature, and the reaction was monitored by TLC. After completion, the mixture was filtered through a sand core funnel, and the filtrate was concentrated under reduced pressure to give the crude product, which was purified by chromatography and eluted with petroleum/ethyl acetate (2:1) to afford the desired compound (0.7 g) as a white solid in 55% yield. ^1^H NMR (400 MHz, CDCl_3_) δ 9.91 (s, 1H), 7.85 (d, *J* = 8.6 Hz, 2H), 7.12 (d, *J* = 8.6 Hz, 2H), 5.60 – 5.41 (m, 2H), 5.17 (d, *J* = 7.8 Hz, 1H), 5.14 (dd, *J* = 10.4, 3.4 Hz, 1H), 4.02 (t, *J* = 6.0 Hz, 1H), 3.55 – 3.46 (m, 2H), 3.34 (s, 3H), 2.18 (s, 3H), 2.05 (s, 3H), 2.01 (s, 3H); ^13^C NMR (101 MHz, CDCl_3_) δ 190.80, 170.21, 170.08, 169.41, 161.49, 131.90, 131.72, 116.73, 98.69, 73.03, 70.88, 70.50, 68.69, 67.51, 59.56, 20.73, 20.67, 20.61.

*3. The synthesis of Compound 3, (2S,3R,4S,5S,6R)-2-(4-(hydroxymethyl)phenoxy)-6-(methoxymethyl)tetrahydro-2H-pyran-3,4,5-triyl triacetate*

Sodium borohydride (100 mg, 3.67 mmol) was added to a stirred solution of 2 (1 g, 2.4 mmol) in MeOH in an ice bath for 10 min, and the reaction was monitored by TLC. After completion, the solution was diluted with acetic ether and washed with saturated ammonium chloride solution and saturated salt water. The organic layer was dried over Na_2_SO_4_ and concentrated under reduced pressure. The resulting crude residue was purified by column chromatography on silica gel and eluted with petroleum/ethyl acetate (1:1) to afford desired compound 3 (0.5 g, 45% yield) as a white solid. ^1^H NMR (400 MHz, CDCl_3_) δ 7.28 (d, *J* = 8.4 Hz, 2H), 6.99 (d, *J* = 8.4 Hz, 2H), 5.54 – 5.39 (m, 2H), 5.10 (dd, *J* = 10.4, 3.4 Hz, 1H), 5.03 (d, *J* = 7.8 Hz, 1H), 4.61 (s, 2H), 3.95 (t, *J* = 6.0 Hz, 1H), 3.54 – 3.43 (m, 2H), 3.32 (s, 3H), 2.16 (s, 3H), 2.04 (s, 3H), 1.99 (s, 3H); ^13^C NMR (101 MHz, CDCl_3_) δ 170.30, 170.16, 169.52, 156.63, 135.79, 128.54, 116.91, 99.75, 72.65, 71.02, 70.49, 68.94, 67.63, 64.76, 59.53, 20.76, 20.70, 20.63.

*4. The synthesis of Compound 4, (2S,3R,4S,5S,6R)-2-(4-(bromomethyl)phenoxy)-6-(methoxymethyl)tetrahydro-2H-pyran-3,4,5-triyl triacetate*

A stirred solution of 3 (1 g, 2.4 mmol) in dry DCM (90 mL) and PBr_3_ (1.2 g, 4.4 mmol) was added into the mixture. The reaction solution was stirred for 2 h in an ice bath. The reaction was monitored by TLC. After the reaction was complete, the solution was diluted with DCM and washed with cold water and saturated salt water. The organic layer was dried over Na_2_SO_4_ and concentrated under reduced pressure. The resulting crude residue was purified by column chromatography and eluted with petroleum/ethyl acetate (3:1) to afford the desired compound 4 (0.9 g, 80% yield) as a white solid. ^1^H NMR (400 MHz, CDCl_3_) δ 7.28 (d, *J* = 8.4 Hz, 2H), 6.99 (d, *J* = 8.4 Hz, 2H), 5.54 – 5.39 (m, 2H), 5.10 (dd, *J* = 10.4, 3.4 Hz, 1H), 5.03 (d, *J* = 7.8 Hz, 1H), 4.85 (s, 2H), 3.95 (t, *J* = 6.0 Hz, 1H), 3.54 – 3.43 (m, 2H), 3.32 (s, 3H), 2.16 (s, 3H), 2.04 (s, 3H), 1.99 (s, 3H); ^13^C NMR (101 MHz, CDCl_3_) δ 170.36, 170.24, 169.39, 156.88, 132.80, 130.50, 117.17, 99.48, 72.65, 71.13, 70.82, 68.60, 66.87, 61.39, 33.14, 20.75, 20.69.

*5. The synthesis of Compound 5, (E)-1-(Pyrrolidin-1-yl)-2-((4-(((2S,3R,4S,5S,6R)-3,4,5-triacetoxy-6-(methoxymethyl)tetrahydro-2H-pyran-2-yl)oxy)benzyl)oxy)diazene oxide*

KI powder (100 mg, 0.6 mmol) was added to a solution of compound 4 (1 g, 2.5 mmol) and diazeniumdiolate sodium (430 mg, 4.1 mmol) in dry DMF (15 mL) in an ice bath. The reaction mixture was stirred under an argon atmosphere at 0°C for 24 h. Then, ice water was added to quench the reaction. The reaction mixture was extracted with ethyl acetate three times, and the combined organic phase was washed with water twice, dried over Na_2_SO_4,_ and concentrated under reduced pressure. The resulting residue was purified by chromatography eluting with petroleum/ethyl acetate (2:1) to afford desirable compound 5 as a pale oil (0.8 g, 60%). ^1^H NMR (400 MHz, CDCl_3_) δ 7.34 (d, *J* = 8.6 Hz, 2H), 6.98 (d, *J* = 8.6 Hz, 2H), 5.54 – 5.43 (m, 2H), 5.11 (s, 2H), 5.04 (d, *J* = 7.8 Hz, 1H), 4.37 – 3.95 (m, 4H), 3.52 – 3.48 (m, 4H), 3.32 (s, 3H), 2.16 (s, 3H), 2.04 (s, 3H), 1.99 (s, 3H), 1.96 – 1.86 (m, 4H); ^13^C NMR (101 MHz, CDCl_3_) δ 170.37, 170.12, 169.40, 157.08, 131.08, 130.35, 116.89, 99.63, 74.62, 72.63, 71.11, 70.85, 68.68, 66.92, 61.40, 50.96, 22.82, 20.75, 20.68.

*6. The synthesis of Compound 6, (E)-1-(Pyrrolidin-1-yl)-2-((4-(((2S,3R,4S,5R,6R)-3,4,5-trihydroxy-6-(methoxymethyl)tetrahydro-2H-pyran-2-yl)oxy)benzyl)oxy)diazene oxide*

## To a solution of compound 5 (500 mg, 1.2 mmol) in dry MeOH (15 mL) was added a catalytic amount of MeONa. The reaction was monitored by TLC after the reaction was completed; the solvent was removed under reduced pressure. The obtained crude product was purified by chromatography using MeOH in DCM (from 3% to 10%) as the eluent to give the desired compound 6 (310 mg) as a white solid with a yield of 80%. ^1^H NMR (400 MHz, DMSO) δ 7.33 (d, *J* = 8.6 Hz, 2H), 7.02 (d, *J* = 8.6 Hz, 2H), 5.06 (s, 2H), 4.88 (d, *J* = 7.8 Hz, 1H), 3.85 – 3.76 (m, 1H), 3.60 – 3.44 (m, 5H), 3.40 (t, *J* = 6.6 Hz, 4H), 3.25 (s, 3H), 1.90 – 1.76 (m, 4H); ^13^C NMR (101 MHz, DMSO) δ 157.86, 130.83, 129.66, 116.44, 100.86, 74.33, 73.73, 73.29, 71.96, 70.44, 68.86, 58.83, 51.05, 22.62.
