# Supplementary material for: Genetically engineered mesenchymal stem cells as a nitric oxide reservoir for acute kidney injury therapy
Source: eLife. 2023 Sep 11;12:e84820. doi: 10.7554/eLife.84820 (PMC10541176; doi:10.7554/eLife.84820)
Supplement: Supplementary file 1. [file elife-84820-supp1.docx]

**Supplementary File 1--** **Antibodies used for Western blotting and Immunostaining analysis**

| Reagent type | Designation | Source or reference | Identifiers | Additional information |
| --- | --- | --- | --- | --- |
| Antibody | Anti-6×His tag  (Rabbit polyclonal) | Abcam | ab9108 | IF:1:200  WB:1:1000 |
| Antibody | Anti-CD31  (Rat monoclonal) | BD | BD-550274 | 1:200 |
| Antibody | Anti-Ki67  (Rat monoclonal) | Invitrogen | AB_10853185 | 1:200 |
| Antibody | Anti-human Ki67  (Mouse monoclonal) | BD | BD-550609 | 1:200 |
| Antibody | Anti-F4/80  (Rat monoclonal) | Abcam | ab16911 | 1:200 |
| Antibody | Anti-iNOS  (Rabbit polyclonal) | Abcam | ab15323 | 1:200 |
| Antibody | Anti-CD206  (Mouse monoclonal) | Santa Cruz Biotechnology | sc-58986 | 1:100 |
| Antibody | Anti-Kim-1  (Rabbit polyclonal) | Abcam | ab47635 | 1:200 |
| Antibody | Anti-α-SMA  (Rabbit polyclonal) | Boster | P62736 | 1:200 |
| Antibody | Anti-COL I  (Rabbit monoclonal) | Abcam | ab260043 | 1:200 |
| Antibody | Anti-Cleaved-caspase-3  (Rabbit polyclonal) | Wanleibio | WL04004 | 1:200 |
