# Supplementary material for: Genetically engineered mesenchymal stem cells as a nitric oxide reservoir for acute kidney injury therapy
Source: eLife. 2023 Sep 11;12:e84820. doi: 10.7554/eLife.84820 (PMC10541176; doi:10.7554/eLife.84820)
Supplement: Supplementary file 2. [file elife-84820-supp2.docx]

# Supplementary File 2. List of human primer sequences used for qPCR analysis in this study.

| **Name** | **Forward** | **Reverse** |
| --- | --- | --- |
| *β-GAL^H363A^* | AACTGCCCCATCTGACAGTG | TCTGCTCTTGAGCAAAGGGG |
| *Rluc* | TCGCCTCCTGGATCACTACA | TCACCTTCACGAACTCGGTG |
| *RFP* | TCCGAGGACGTCATCAAGGA | TGTCCAGCTTGATGTCGGTC |
| *VEGFA* | AAGCGCAAGAAATCCCGGTA | CGCGAGTCTGTGTTTTTGCA |
| *FGF-2* | GCTGTACTGCAAAAACGGGG | AGCCAGGTAACGGTTAGCAC |
| *HGF* | ACCCTGGTGTTTCACAAGCA | GCAAGAATTTGTGCCGGTGT |
| *IGF-1* | GCTGGTGGATGCTCTTCAGT | TTGAGGGGTGCGCAATACAT |
| *ANG-1* | AGCAACTGGAGCTGATGGAC | TTACAGTCCAACCTCCCCCA |
| *IL-10* | CCAGCTTGAGAACAGCTGCA | CGCCTTGATGTCTGGGTCTT |
| *IL-1β* | CGGCATCCAGCTACGAATCT | TGGACCAGACATCACCAAGC |
