# Supplementary material for: Genetically engineered mesenchymal stem cells as a nitric oxide reservoir for acute kidney injury therapy
Source: eLife. 2023 Sep 11;12:e84820. doi: 10.7554/eLife.84820 (PMC10541176; doi:10.7554/eLife.84820)
Supplement: Supplementary file 3. [file elife-84820-supp3.docx]

# Supplementary File 3. List of mouse primer sequences used for qPCR analysis in this study.

| **Name** | **Forward** | **Reverse** |
| --- | --- | --- |
| Vegfr2 | ACACGGTCATCCTCACCAAC | TTGGCGTAGACTGTGCATGT |
| Fgf-2 | GCCAACCGGTACCTTGCTAT | GTCCCGTTTTGGATCCGAGT |
| Plgf | GTAGTGGCTGCTGTGGTGAT | TGGGGTTCCTCAGTCTGTGA |
| Ang-1 | CTCTGCAAAGGGATGCTCCA | TATCTCAAGCATGGTGGCCG |
| Ang-2 | GGGACGACAGATACTGCGAA | TAGAGGCTCGGTACCTGCAT |
| iNOS | CCAAGCCCTCACCTACTTCC | CTCTGAGGGCTGACACAAGG |
| Arg-1 | CTCCAAGCCAAAGTCCTTAGAG | GGAGCTGTCATTAGGGACATCA |
| IL-1β | CAACCAACAAGTGATATTCTCCATG | GATCCACACTCTCCAGCTGCA |
| IL-4 | CTCAACCCCCAGCTAGTTGT | TGCATGATGCTCTTTAGGCT |
| Col1A1 | ACTTCACTTCCTGCCTCAG | TGACTCAGGCTCTTGAGGGT |
| Fibronectin | ATGTGGACCCCTCCTGATAGT | GCCCAGTGATTTCAGCAAAGG |
| TGF-β | GTCAGACATTCGGGAAGCAG | GCGTATCAGTGGGGGTCA |
| MMP-2 | ACAACAGCTGTACCACCGAG | GGATCTGAGCGATGCCATCA |
| MMP-9 | GTCTTCCTGGGCAAGCAGTA | CTGGACAGAAACCCCACTTC |
| TIMP-1 | TGGCATCTGGCATCCTCTTG | CAAGCAAAGTGACGGCTCTG |
| TIMP-2 | TGAGCGAGAAGGAGGTGGAT | CGGGTCCTCGATGTCAAGAA |
| BMP-7 | TCCACCCTCGATACCACCAT | GCTGTTTTCTGCCACACTGG |
